# Genicular Artery Embolization in Moderate to Severe Knee Osteoarthritis: Technique, Safety and Clinical Outcome

**DOI:** 10.1007/s00270-025-03983-2

**Published:** 2025-02-19

**Authors:** A. Taheri Amin, I. Frommhold, A. Huebner, M. Boschheidgen, M. Frenken, K. Jannusch, E. Tietz, D. Weiss, L. M. Wilms, F. Ziayee, P. Minko

**Affiliations:** Department of Diagnostic and Interventional Radiology, Medical Faculty, University Dusseldorf, Moorenstrasse 5, 40225 Dusseldorf, Germany

**Keywords:** Genicular artery embolization, Technique, Safety, Clinical outcome, Radiation exposure, Vascular complications

## Abstract

**Purpose:**

To evaluate the safety and clinical outcomes of genicular artery embolization for knee osteoarthritis (OA).

**Materials and Methods:**

A total of 43 patients suffering from osteoarthritis of the knee (Kellgren and Lawrence grades 2–4) were included in this prospective study. Peri-interventional data including vascular access, embolized target vessels, fluoroscopy time and radiation dose were collected. After 2-, 3- and 12-month pain scores, functional outcomes and adverse events were assessed through a standardized questionnaire.

**Results:**

All embolizations were performed via a coaxial system consisting of a 4F Cobra catheter and a Microcatheter without the use of an introducer sheath. A mixture of contrast agent (Accupaque, GE, USA) and microspheres (Embosphere, Merit Medical, USA) was injected. At least three genicular branches were embolized per patient with following incidence: inferior lateral genicular artery (77%), superior lateral genicular artery (74%) and descending genicular artery (74%). The mean total volume of permanent embolic agent used was 3.6 ± 1.3 ml. The average fluoroscopy time was 29 ± 11 min, and radiation dose was 40.84 ± 26.21 Gy/cm^2^. During the 1-year follow-up, patients pain while walking showed an average reduction of 2.0 ± 0.5 points on the numeric rating scale (*p* < 0.0001), without any significant difference between different grades of osteoarthritis. Besides mild transient skin discolorations in four patients, no complications were observed.

**Conclusion:**

Embolization of multiple genicular artery branches in a single session using microspheres in averaged doses higher than 2 ml total is safe and effective in reducing pain and improving functionality in patients with symptomatic OA, regardless of severity grade.

**Graphical Abstract:**

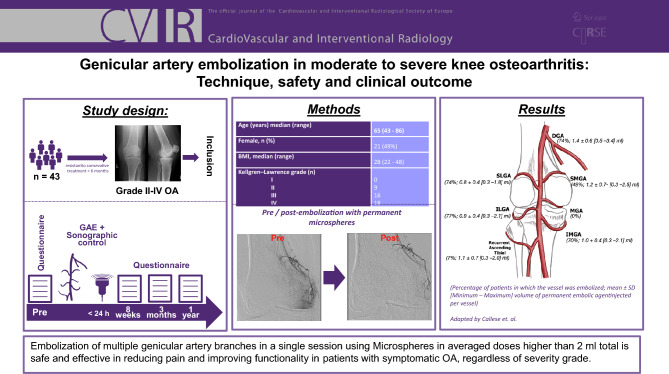

## Introduction

Knee osteoarthritis (OA) is the most prevalent joint disorder, significantly impacting individuals' quality of life due to pain and restricted mobility [1]. There are a number of pharmacological and non-pharmacological interventions for mild to moderate OA, but these rarely lead to a noticeable reduction in pain and do not modify the course of the disease [2]. In recent years, genicular artery embolization (GAE) has emerged as a promising option for patients with knee pain due to OA refractory to conservative therapies.

GAE is a minimally invasive procedure that targets the neovascularization associated with knee OA [3]. The effectiveness of GAE has been demonstrated in several clinical studies, with a growing body of evidence suggesting that it leads to significant and sustained pain reduction, thereby improving the functionality and quality of life for patients with knee OA [3–11]. However, there are still several unresolved issues, particularly regarding the technical aspects of the procedure.

The choice of embolic agent, the amount applied per genicular artery branch and the number of branches embolized in one session still vary greatly between different studies [12–16]. With more interventions being done in an outpatient setting, patient safety and cost efficiency has become more important.

Another gap in the current literature is the lack of systematic studies focusing on patients with severe OA. While some studies have included such patients [6, 8, 10, 11, 17], only two studies have compared the outcome after GAE between different OA grades [8, 18] partly contradicting the findings of systematic reviews and meta-analysis [13, 14].

The aim of this study is to investigate the effectiveness and safety of GAE in patients with moderate to severe OA in an outpatient setting, embolizing at least three genicular branches.

## Materials and Methods

### Study Design

The study was conducted at the University Hospital of Duesseldorf as a prospective, single-center and non-randomized clinical investigation. Eligible participants were 18–90 years of age, had signs of OA on radiographs and moderate to severe knee pain that was resistant to at least 6 months of conservative treatment. Exclusion criteria included pregnancy, severe peripheral artery disease (Rutherford grade III or higher), active or suspected knee infection, irreversible coagulopathy or a bleeding diathesis or renal impairment (eGFR < 45). Before the intervention, radiographs of the knee were reviewed by three experienced radiologists (L.W., M.B. and K.J.), and OA was graded using the Kellgren–Lawrence scale (K&L) as doubtful (K&L I), mild (K&L II), moderate (K&L III) and severe (K&L IV).

This study was approved by the Institutional Review Board (IRB) and conducted in accordance with the Declaration of Helsinki. Informed consent was obtained from all participants before the study.

### Procedure

**Fig. 1:**
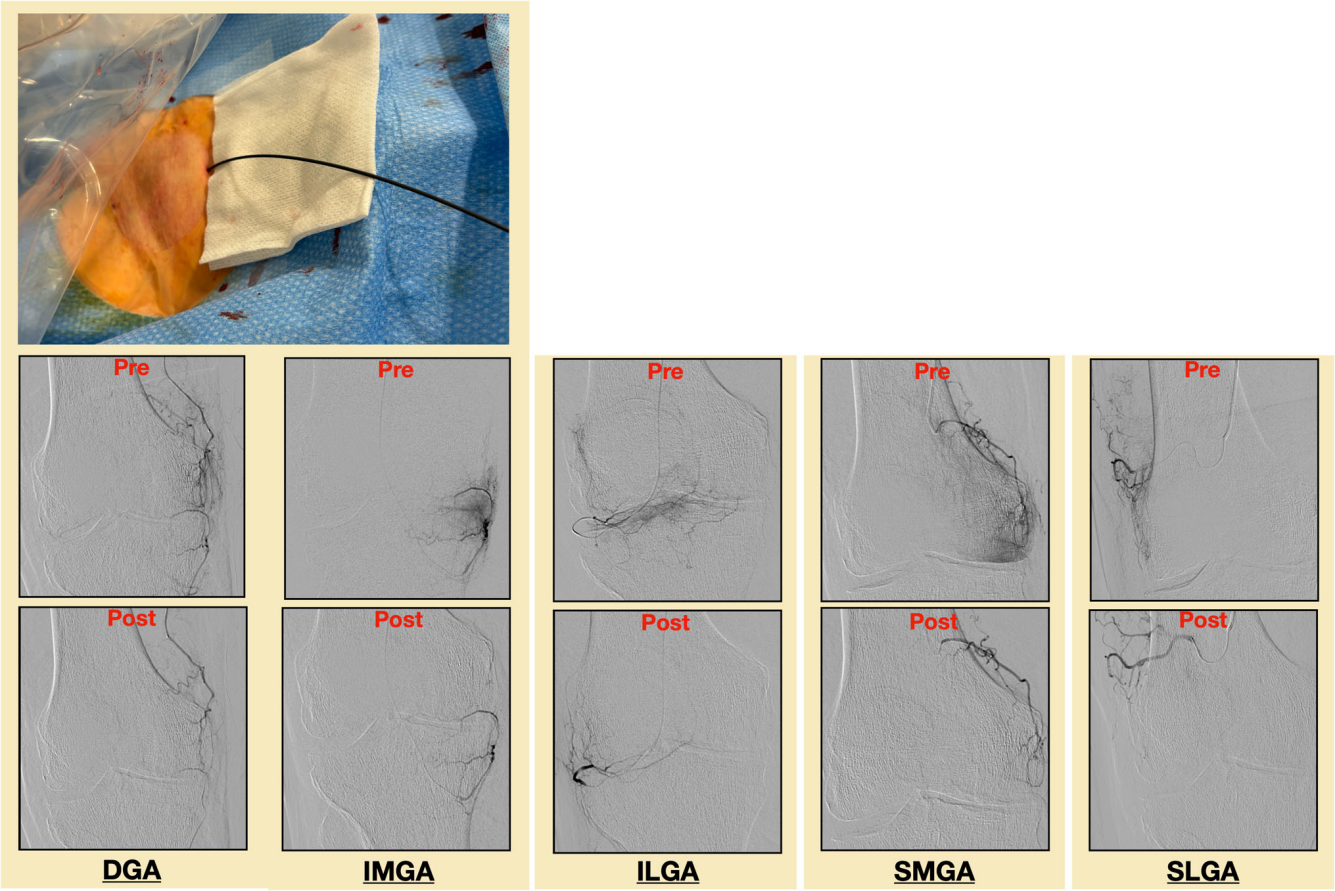
Case example. Embolization in a 78-year-old male suffering from KL-Grade 4 osteoarthritis via an antegrade transfemoral access with a coaxial system consisting of a 4F Cobra catheter and a 1.7 F microcatheter without the use of an introducer sheath. Extensive vascular blush was seen in the descending genicular artery (DGA), inferior medial genicular artery (IMGA) and inferior lateral genicular artery (ILGA). The superior medial genicular artery (SMGA) and the superior lateral genicular artery (SLGA) showed only minimal vascular blush and were embolized in the same session

All interventions were performed by two interventional radiologists each with over 15 years of experience (P.M. and F.Z.). Transfemoral vascular access was gained via an ipsilateral antegrade (*n* = 37) or contralateral cross-over (*n* = 6) approach without the use of an introducer sheath. A contralateral approach was chosen in obese patients (BMI > 25 kg/m^2^) to achieve a stable access and reduce risk of possible access site complications. A 4F Cobra catheter (Merit Medical, USA) was used to gain access to the distal superficial femoral artery, from where angiography was performed using 300 mg/ml iodinated contrast (Accupaque, GE HealthCare, USA). After visualization of the genicular arterial anatomy, selective canalization of the genicular arteries was performed using a 1.7 F microcatheter (Merit Medical, USA). Only genicular branches showing a hyperaemic blush were embolized. Prior to injection, the permanent embolic agent (100–300 μm Embospheres, Merit Medical, USA) was diluted in 10 mL of iodinated contrast agent (Accupaque 300 mg/mL, GE HealthCare, USA). During injection, aliquots of the mixture were injected into the target vessels to “prune” the abnormal neovessels while maintaining the normal inflow of the parenting vessel. Ice packs were placed around the knee joint to reduce nontarget cutaneous ischemia and postprocedural skin discoloration by promoting temporary cutaneous vasoconstriction. Post-procedure, patients were observed in our outpatient clinic for at least 4 h before discharge, Fig. 1.

Technical success was defined as selective catheterization and embolization of at least one genicular artery. Arteries treated, volume of permanent embolic agent used, total radiation dosage and time were recorded in a standardized report.

**Fig. 2:**
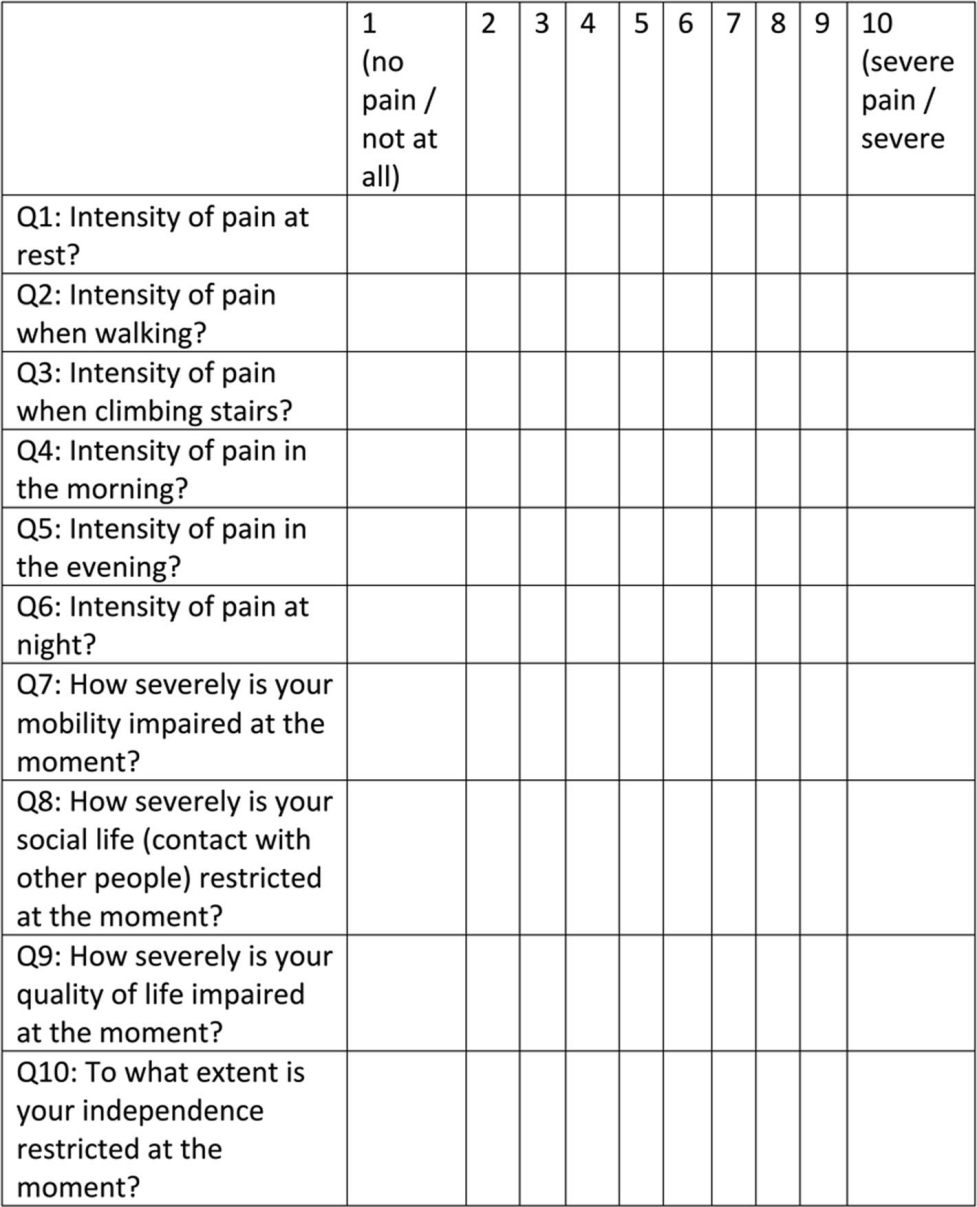
English translation of the patient questionnaire

Clinical outcome was assessed by a self-developed 10-item questionnaire that measures pain (6 items), functionality (2 items) and quality of life (2 items) using a numerical rating scale (Fig. 2). Patients were asked to fill out the questionnaire before and 2, 3 and 12 months after the intervention. Any reduction in pain and/or improvement of functionality/quality of life was considered as a clinical success. Vascular and non-vascular complications were checked upon discharge, 24 h after embolization with duplex/doppler ultrasound as well as 2, 3 and 12 months during follow-up visits clinically and reported in line with the Cardiovascular and Interventional Radiological Society of Europe (CIRSE) Quality Assurance Document and Standards for Classification of Complications [19]. Figure 1 shows a case in which five genicular branches were embolized in one session.

### Statistical Analysis

Data analysis included descriptive statistics as well as comparison of outcome between the different time points and the different grades of OA using a mixed model analysis of variance. Statistical analysis was performed using PRISM-GraphPad® version 10. A *p* value of < 0.05 was considered statistically significant.

The sample size was calculated using G*Power. Based on a significance level of *α* = 0.05 and a power of 1 − *β* = 0.80, the required sample size was determined to detect a medium effect size (Cohen's *d* = 0.5) difference between patients before and after GAE. The calculation took into account drop-out rates. A total of 40 participants were deemed necessary to achieve adequate statistical power for the primary outcome of the study, which was a reduction in the numeric pain rating scale (NRS).

## Results

A total of 43 patients (female: *n* = 21) with a median age of 65 years (± 11; [43–86] years) and a median BMI of 28 kg/m^2^ (± 5; [23–48] kg/m^2^) were included in the study (Table 1). Most patients suffered from moderate (*n* = 16; 37%) or severe (*n* = 18; 42%) OA while the rest showed mild sign of OA.

**Table 1:** Patient characteristics at baseline and follow-up

	Baseline	8 weeks	3 months	12 months
Number of patients (*n*)	43	43	32	38
Age (years) median (range)	65 (43–86)	65 (43–86)	67 (43–86)	65 (43–86)
Female, *n* (%)	21 (49%)	21 (49%)	15 (46%)	19 (50%)
BMI, median (range)	28 (22–48)	28 (22–48)	28 (22–39)	28 (23–48)
Kellgren–Lawrence grade (*n*)				
I	0	0	0	0
II	9	9	6	8
III	16	16	13	14
IV	18	18	13	16

**Fig. 3:**
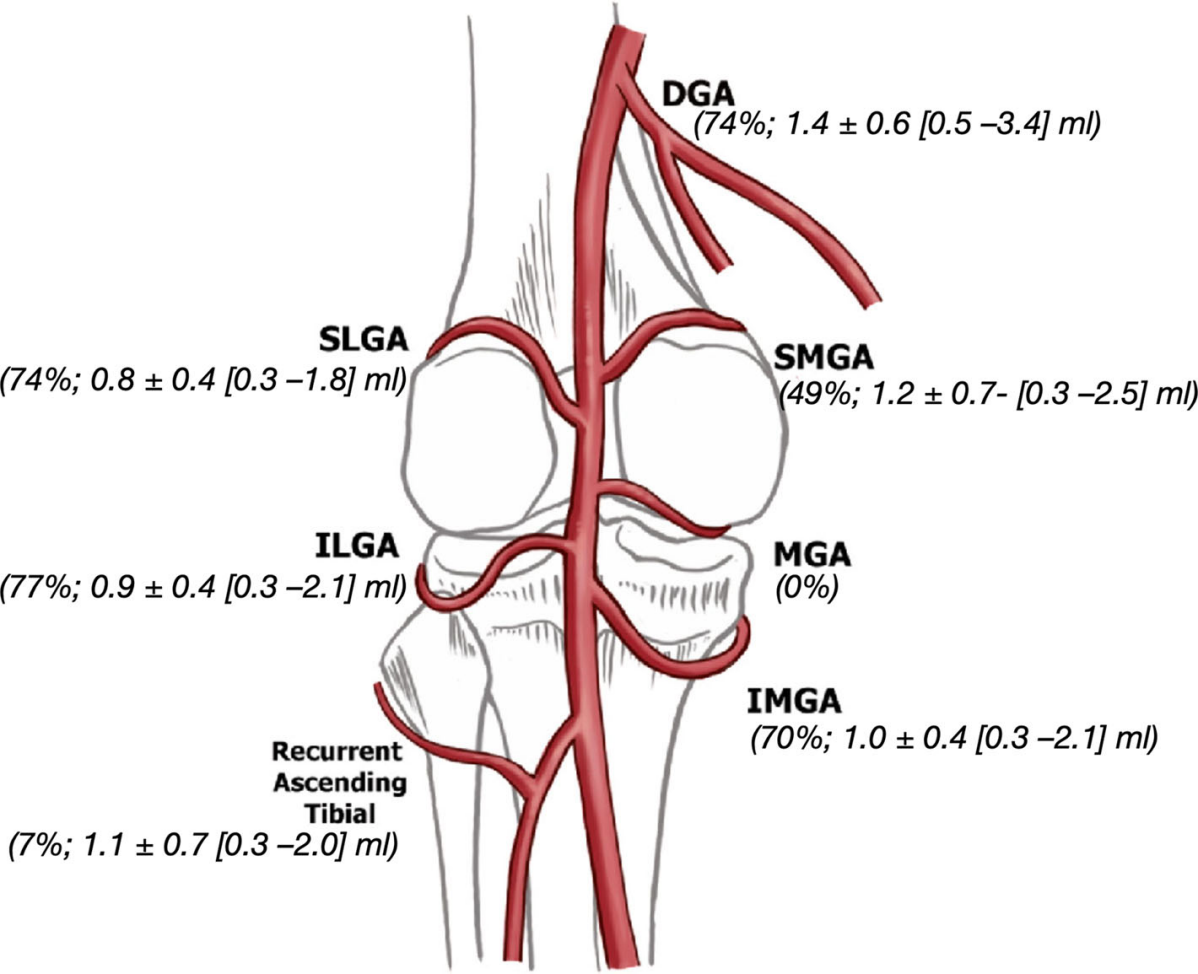
Technical procedural parameters. Schematic of the genicular arteries adapted by Callese et al. [30]. DGA: Descending genicular artery; SLGA: Superior lateral genicular artery; ILGA: Inferior genicular artery; SMGA: Superior medial genicular artery; IMGA: Inferior medial genicular artery and MGA: Middle genicular artery. (Percentage of patients in which the vessel was embolized; mean ± SD [Minimum–Maximum] volume of permanent embolic agent injected per vessel)

Technical success was achieved in all patients (*n* = 43). At least three genicular branches were embolized in one session (Fig. 3) with the inferior lateral genicular artery (ILGA; *n* = 33; 77%) being the most common, followed by the descending genicular artery (DGA; *n* = 32; 74%), the superior lateral genicular artery (SLGA; *n* = 32; 74%), the inferior medial genicular artery (IMGA; *n* = 30; 71%) and the superior medial genicular artery (SMGA; *n* = 21; 49%). The recurrent ascending tibial artery was embolized in 7% of patients (ARTA; *n* = 3; 7%). In 22 patients, three and, in 15 patients, four genicular branches were embolized in one session, while in six patients, five genicular branches were embolized.

The mean total volume of permanent embolic agent used was 3.6 ml (± 1.3; [1.3–6.8] ml). The mean volume administered in the DGA was 1.4 ml (± 0.6 [0.5–3.4] ml) followed by the SMGA (1.2 ml ± 0.7; [0.3–2.5] ml), ARTA (1.1 ± 0.7 [0.3–2.0] ml), IMGA (1.0 ± 0.4 [0.3–2.1] ml), ILGA (0.9 ± 0.4 [0.3–2.1] ml) and the SLGA (0.8 ± 0.4 [0.3–1.8] ml).

The mean fluoroscopy time was 29 min (± 11; [10–59] min), and average radiation dose was 40.84 Gy/cm^2^ (± 26.21; [5.6–118.4] Gy/cm^2^). In four patients, mild skin discolorations were observed 24 h after the intervention, all of which resolved completely during the 2-month follow-up. No other vascular or non-vascular complications were reported.

Forty-three patients (100%) reported outcomes during the first follow-up (1 FU) after 2 months. Thirty-two patients (74%) completed the second follow-up (2 FU) after 3 months, and 38 patients (88%) completed the third follow-up (3 FU) after 12 months. Despite technically and clinically successful GAE, five patients (OA grade II: *n* = 1; grade III: *n* = 2 and OA grade IV: *n* = 2) did not gain satisfactory pain reduction and opted for joint replacement at a mean of 8 months (± 2 [4–11] months) following GAE.

**Fig. 4:**
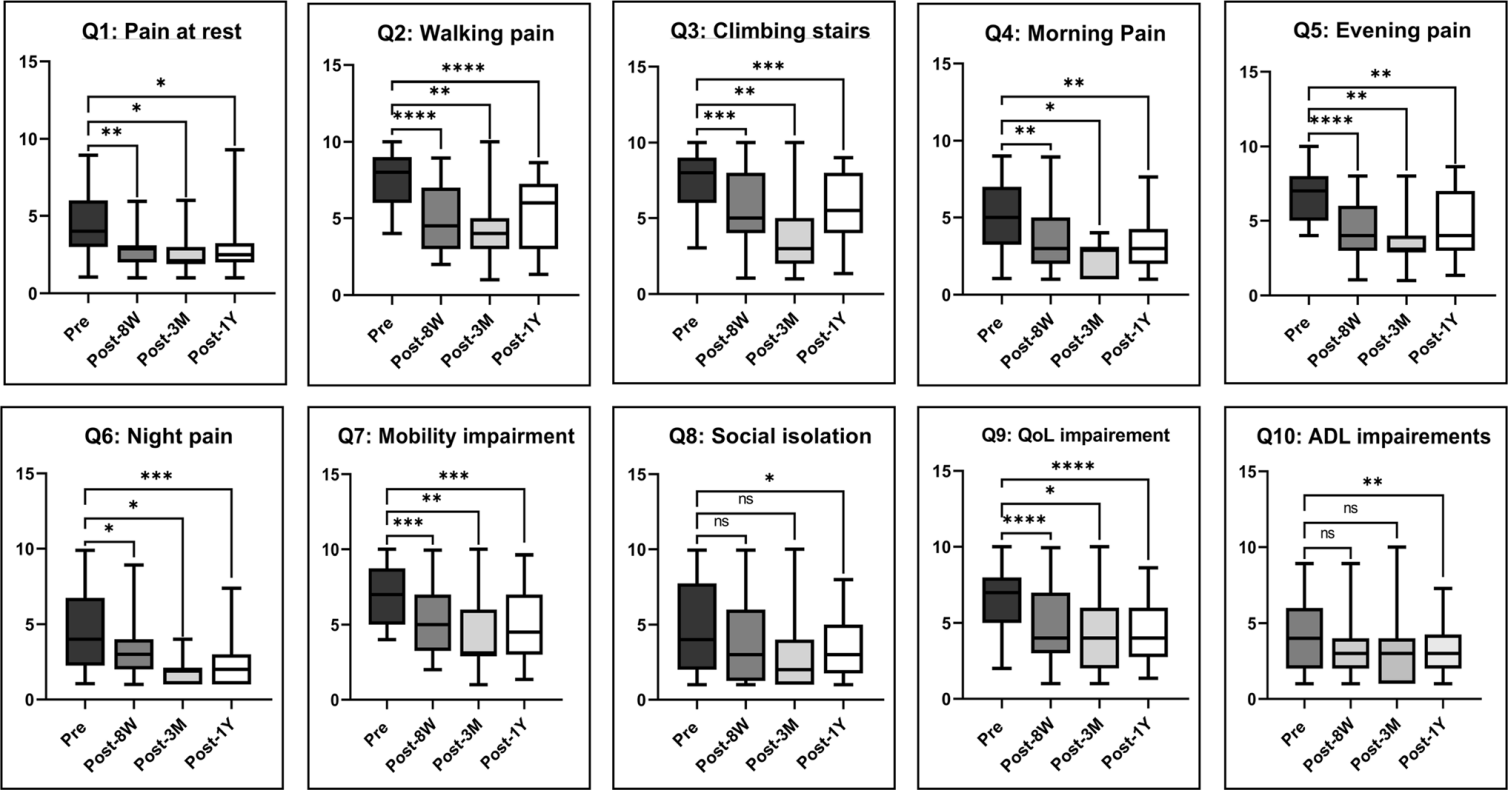
Changes in pain and function after GAE. QoL: Quality of life and ADL: Activities of daily living; ns: *p* > 0.05; *p = 0.01–0.05; **p = 0.001–0.01; ***p = 0.0001–0.001 and ****p < 0.0001

All items quantifying pain (Item 1–6) showed significant reduction compared to baseline during every follow-up visit (Fig. 4 and Table 2) with mean reduction between baseline and 1-year follow-up ranging between 1.3 points (95% CI 0.2–2.4) for “pain at rest” and 2.1 points (95% CI 1.1–3.1) for “walking pain.”

**Table 2:** Patient questionnaire outcomes overall

Assessment	Visit	Mean NRS	*p* value
Pain at rest	Baseline	4.4	
	8 weeks	2.9	< 0.01
	3 months	2.1	< 0.05
	12 months	3.3	< 0.05
Pain while walking	Baseline	7.4	
	8 weeks	5.0	< 0.0001
	3 months	4.1	< 0.01
	12 months	4.8	< 0.0001
Pain while climbing stairs	Baseline	7.6	
	8 weeks	5.8	< 0.001
	3 months	4.3	< 0.01
	12 months	4.8	< 0.001
Morning pain	Baseline	5.5	
	8 weeks	3.9	< 0.01
	3 months	2.8	< 0.05
	12 months	3.2	< 0.01
Evening pain	Baseline	6.7	
	8 weeks	4.2	< 0.0001
	3 months	3.3	< 0.01
	12 months	4.2	< 0.01
Night pain	Baseline	4.4	
	8 weeks	3.2	< 0.05
	3 months	2.3	< 0.05
	12 months	2.9	< 0.001
Mobility impairment	Baseline	6.9	
	8 weeks	5.2	< 0.001
	3 months	4.1	< 0.01
	12 months	4.4	< 0.001
Social isolation	Baseline	4.7	
	8 weeks	4.1	ns
	3 months	4.0	ns
	12 months	3.7	< 0.05
QoL impairment	Baseline	6.5	
	8 weeks	4.8	< 0.0001
	3 months	3.9	< 0.05
	12 months	4.4	< 0.0001
ADL impairments	Baseline	4.3	
	8 weeks	3.4	ns
	3 months	3.8	ns
	12 months	3.2	< 0.01

QoL, Quality of life; ADL, Activities of daily living and NRS, Numeric rating scale

Mobility impairments (Item 7) showed significant reduction during all follow-up visits with a mean reduction of 1.9 points (95% CI 0.8–3.0) between baseline and 1-year follow-up.

A significant difference in Item 8, measuring social isolation, was only seen, when comparing baseline and 1-year follow-up with a mean reduction of 1.2 points (95% CI 0.2–2.2). Similarly, Item 10, measuring disability in activities of daily living (ADL disability), also showed significant differences only, when comparing baseline and 1-year follow-up with a mean reduction of 1.2 points (95% CI 0.3–2.2).

Quality of life (Item 9) improved during all follow-up visits compared to baseline with a mean difference of 2.3 points (95% CI 1.2–3.3) between baseline and 1-year follow-up.

**Fig. 5:**
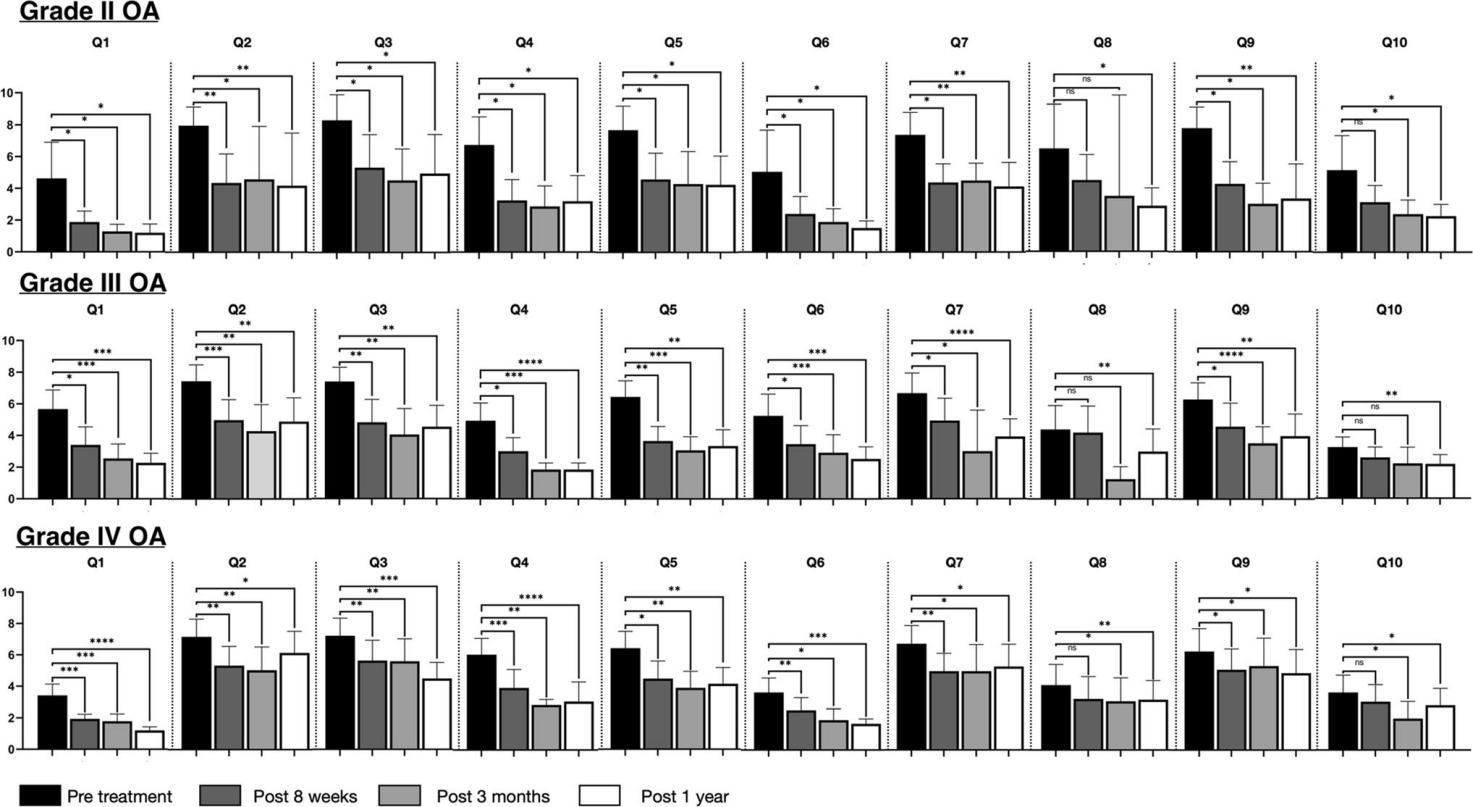
Changes in pain and function after GAE for OA grades II, III and IV. Q1: Pain at rest; Q2: Waking pain; Q3: Pain while climbing stairs; Q4: Morning pain; Q5: Evening pain; Q6: Night pain; Q7: Mobility impairment; Q8: Social isolation; Q9: Quality of life impairment and Q10 Activities of daily living impairments; ns: *p* > 0.05; *: *p* = 0.01–0.05; **p = 0.001–0.01; ***p = 0.0001–0.001 and ****p < 0.0001

Patients with mild, moderate and severe radiographic OA all showed significant reduction in the numeric rating scale (NRS) for all items of the questionnaire when compared to baseline during follow-up visits (Fig. 5 and Table 3). For Item 8 (social isolation) and Item 10 (ADL disability), a significant reduction between baseline and follow-up was only seen at 12 months for patients with grades II and III OA, while patients with grade IV OA showed significant reduction in Items 8 and 10 beginning at the 3-month follow-up.

**Table 3:** Patient questionnaire outcomes overall

Assessment	Visit	Grade II OA		Grade III OA		Grade IV OA	
		Mean NRS	*p* value	Mean NRS	*p* value	Mean NRS	*p* value
Pain at rest	Baseline	4.4		5.5		3.3	
	8 weeks	1.7	< 0.05	3.4	< 0.05	1.8	< 0.001
	3 months	1.1	< 0.05	2.5	< 0.001	1.6	< 0.001
	12 months	1.5	< 0.05	2.8	< 0.001	1.4	< 0.0001
Pain while walking	Baseline	8.0		7.3		7.3	
	8 weeks	4.7	< 0.01	4.9	< 0.001	5.3	< 0.01
	3 months	6.0	< 0.05	4.9	< 0.001	4.3	< 0.01
	12 months	4.2	< 0.01	4.4	< 0.01	4.8	< 0.05
Pain while climbing stairs	Baseline	8.7		7.3		7.2	
	8 weeks	6.1	< 0.05	4.8	< 0.01	5.3	< 0.01
	3 months	5.7	< 0.05	4.2	< 0.01	3.7	< 0.01
	12 months	6.0	< 0.05	4.5	< 0.01	3.8	< 0.001
Morning pain	Baseline	6.6		4.8		5.5	
	8 weeks	3.6	< 0.05	2.9	< 0.05	2.3	< 0.001
	3 months	2.7	< 0.05	2.5	< 0.001	2.8	< 0.01
	12 months	3.5	< 0.05	1.6	< 0.0001	2.3	< 0.0001
Evening pain	Baseline	7.4		6.3		6.6	
	8 weeks	4.4	< 0.05	3.8	< 0.01	4.4	< 0.05
	3 months	5.3	< 0.05	2.9	< 0.001	3.7	< 0.01
	12 months	5.7	< 0.05	3.3	< 0.01	2.9	< 0.01
Night pain	Baseline	4.9		5.1		3.6	
	8 weeks	2.4	< 0.05	3.6	< 0.05	1.9	< 0.01
	3 months	2.1	< 0.05	2.4	< 0.001	2.0	< 0.05
	12 months	2.1	< 0.05	2.1	< 0.001	2.5	< 0.001
Mobility impairment	Baseline	7.3		6.5		6.9	
	8 weeks	4.7	< 0.05	5.0	< 0.05	4.4	< 0.01
	3 months	3.8	< 0.01	4.9	< 0.05	4.5	< 0.05
	12 months	3.0	< 0.01	2.8	< 0.0001	4.6	< 0.05
Social isolation	Baseline	6.6		4.4		4.0	
	8 weeks	4.8	ns	4.1	ns	3.3	ns
	3 months	3.7	ns	3.1	0.0739	2.8	< 0.05
	12 months	2.4	< 0.05	2.9	< 0.01	2.1	< 0.01
QoL impairment	Baseline	7.7		6.3		6.1	
	8 weeks	4.9	< 0.05	4.8	< 0.05	4.9	< 0.05
	3 months	4.8	< 0.05	3.7	< 0.0001	4.3	< 0.05
	12 months	2.9	< 0.01	4.4	< 0.01	4.3	< 0.05
ADL impairments	Baseline	5.0		4.6		3.6	
	8 weeks	3.2	ns	4.0	ns	3.0	ns
	3 months	2.3	< 0.05	4.1	0.6479	2.2	< 0.05
	12 months	3.0	< 0.05	2.4	0.0027	2.2	< 0.05

QoL, Quality of life; ADL, Activities of daily living and NRS, Numeric rating scale

For patients with grade II OA, the mean reduction of the NRS ranged between 2.0 (95% CI 0.9–4.9) for question 10 (impairment in activities of daily living) and 4.8 (95% CI 1.9–7.6) for question 9 (restriction of the quality of life), patients with grade III OA ranged between 1.7 (95% CI 0.4–3.1) for question 9 (restriction of the quality of life) and 3.7 (95% CI 0.5–6.8) for question 7 (mobility impairment) and patients with grade IV OA ranged between 1.1 (95% CI 0.3–1.9) for question 6 (night pain) and 3.2 (95% CI 1.6–4.8) for question 4 (morning pain).

## Discussion

In this study, GAE was shown to significantly reduce pain and improve quality of life and functionality in patients with both mild to moderate and severe OA. The reduction in pain was already measurable early after the intervention, while functionality and quality of life show significant improvements with a delay of about 1 year owing to the more complex and multifactorial nature of these outcome parameters.

The efficacy of GAE using a permanent embolic agent in patients with grades II and III OA has already been investigated in multiple studies [4, 5, 7, 9], including long-term studies [4, 7, 20]. In these studies, visual analog scales (VAS) were predominantly used instead of numerical scales as in this study. Similar to this study however, patients with grades II–III OA showed a significant reduction in pain with sustained efficacy at 1-year follow-up.

Patients with severe OA have been included in only five clinical trials to date [6, 8, 10, 11, 18], of which only two used permanent embolic agents [10, 11]. Studies using temporary embolic agents have shown that grade IV OA is a predictor of poor treatment response [6, 18] and that pain initially decreases after GAE but increases to baseline levels within 3 months [8]. Padia et al. and Sun et al. investigated outcome after GAE with permanent embolic agent in patients with grade II–IV OA. Both studies found that there was a significant reduction in pain, with sustained reduction at 6-month and 1-year follow-up, respectively. Subgroup analyses that compared the outcome between the individual OA grades were not carried out. However, it can at least be hypothesized that the choice of embolic agent influences the outcome of GAE, in patients with grade IV OA.

At an average of 3.6 ± 1.3 mL, the amount of permanent embolic agent applied per session in this study is markedly higher than the amount of 2 mL defined as “common” by the Society of Interventional Radiology [21]. In this study, the embolic agent was diluted with only 10 mL of contrast medium, whereas in most other studies, larger dilutions of up to 20 mL [9] were used. The average number of genicular branches embolized in one session in this study was also higher than in most other studies. One possible factor contributing to the higher amount of permanent embolic agent used in this study may result from the higher percentage of patients with grade IV OA included in this study. Based on the chronically progressive nature of OA, the hypothesis can be made, that as the degree of OA increases, the angiogenesis targeted by GAE also increases [22, 23], making larger amounts of embolic agent necessary. Other than transient skin discolorations in four patients, the use of higher amounts of permanent embolic agent did not lead to an increase of vascular or non-vascular complications. All embolizations were performed without the use of an introducer sheath, which compared to other studies using an introducer sheath [4, 6, 8–11, 24, 25] did not lead to an increase in fluoroscopy time, radiation time and exposure [14]. The feasibility and safety of a transradial and transfemoral sheathless approach for common body interventions have been demonstrated in several studies [26–29]. With growing popularity of GAE and the increasing trend toward outpatient procedures in general not only the safety but also the cost efficiency of interventional procedures will become more important. In this context, a single vascular sheeth may appear to be a minor expense when considered individually. However, as the number of interventions increases, these costs accumulate and the waiver of a vascular sheath may enable cost savings.

A major limitation of this study is the use of a non-validated questionnaires, which was initially chosen to increase patients’ compliance. Other limitations include the lack of a control group and a lack of matching between the different OA grades. Furthermore, other potentially confounding supportive interventions such as pain medication or steroid injections pre- and post-GAE were not analyzed.

The reduced follow-up rate at 3 months can partly be explained by the noticeable improvement in symptoms most patients experience shortly after GAE, which may reduce their willingness to report back on the follow-up visits. At 12 months, this problem was recognized, and patients were contacted more actively. This contributes to the lower level of statistical significance of the 2-month follow-up data when compared to the other time points.

In conclusion, performing GAE with high amounts of permanent embolic agent and routinely embolizing more than two genicular branches in one session can considered to be safe and may even be necessary in patients with grade IV OA in order to achieve long-lasting reduction of pain symptoms. Waving of a vascular sheath can increase the cost-effectiveness of the procedure, without prolonging fluoroscopy time and radiation exposure, increasing patient safety. Based on these results, future studies should not exclude patients with grade IV OA, but investigate intervention techniques that may improve the outcomes in this patient group.
